# Molecular subtype-specific efficacy of anti-EGFR therapy in colorectal cancer is dependent on the chemotherapy backbone

**DOI:** 10.1038/s41416-021-01477-9

**Published:** 2021-07-12

**Authors:** Sanne ten Hoorn, Dirkje W. Sommeijer, Faye Elliott, David Fisher, Tim R. de Back, Anne Trinh, Lianne Koens, Tim Maughan, Jenny Seligmann, Matthew T. Seymour, Phil Quirke, Richard Adams, Susan D. Richman, Cornelis J. A. Punt, Louis Vermeulen

**Affiliations:** 1 Amsterdam UMC, University of Amsterdam, LEXOR, Center for Experimental and Molecular Medicine, Cancer Center Amsterdam, Amsterdam, The Netherlands; 2 Oncode Institute, Amsterdam UMC, Amsterdam, The Netherlands; 3 Department of Medical Oncology, Amsterdam UMC, University of Amsterdam, Amsterdam, The Netherlands; 4 Flevohospital, Department of Internal Medicine, Almere, The Netherlands; 5 Leeds Institute of Medical Research at St James’s, University of Leeds, St James’s University Hospital, Leeds, UK; 6 MRC Clinical Trials Unit, University College London, London, UK; 7 Department of Medical Oncology, Dana-Farber Cancer Institute, Boston, MA USA; 8 Department of Medicine, Harvard Medical School, Boston, MA USA; 9 Department of Pathology, Amsterdam UMC, University of Amsterdam, Amsterdam, The Netherlands; 10 Department of Oncology, University of Oxford, Oxford, UK; 11 Centre for Trials Research Cardiff University and Velindre Hospital, Cardiff, Wales UK; 12 Department of Epidemiology, Julius Center for Health Sciences and Primary Care, University Medical Center, Utrecht University, Utrecht, The Netherlands

**Keywords:** Colorectal cancer, Chemotherapy, Metastasis

## Abstract

**Background:**

Patient selection for addition of anti-EGFR therapy to chemotherapy for patients with *RAS* and *BRAF* wildtype metastatic colorectal cancer can still be optimised. Here we investigate the effect of anti-EGFR therapy on survival in different consensus molecular subtypes (CMSs) and stratified by primary tumour location.

**Methods:**

Retrospective analyses, using the immunohistochemistry-based CMS classifier, were performed in the COIN (first-line oxaliplatin backbone with or without cetuximab) and PICCOLO trial (second-line irinotecan with or without panitumumab). Tumour tissue was available for 323 patients (20%) and 349 (41%), respectively.

**Results:**

When using an irinotecan backbone, anti-EGFR therapy is effective in both CMS2/3 and CMS4 in left-sided primary tumours (progression-free survival (PFS): HR 0.44, 95% CI 0.26–0.75, *P* = 0.003 and HR 0.12, 95% CI 0.04–0.36, *P* < 0.001, respectively) and in CMS4 right-sided tumours (PFS HR 0.17, 95% CI 0.04–0.71, *P* = 0.02). Efficacy using an oxaliplatin backbone was restricted to left-sided CMS2/3 tumours (HR 0.57, 95% CI 0.36–0.96, *P* = 0.034).

**Conclusions:**

The subtype-specific efficacy of anti-EGFR therapy is dependent on the chemotherapy backbone. This may provide the possibility of subtype-specific treatment strategies for a more optimal use of anti-EGFR therapy.

## Introduction

Since the introduction of targeted agents, anti-VEGF and anti-EGFR therapy have become part of the standard treatment arsenal for patients with metastatic colorectal cancer (mCRC). Anti-EGFR agents (cetuximab, panitumumab) may be given as monotherapy in chemorefractory patients, but are usually combined in earlier lines (first or second line) with chemotherapy, with either an oxaliplatin or irinotecan backbone, which are both considered effective and safe regimens [1, 2].

The efficacy of anti-EGFR therapy was shown to be restricted to the subgroup of patients with *RAS* or *BRAFV600E* wildtype tumours, as no benefit was observed in patients with tumours that harbour these mutations [3, 4]. Anti-EGFR therapy was even associated with a detrimental effect in patients with *KRAS* mutant tumours [5–7]. Patient selection for anti-EGFR therapy was further improved by taking into account the sidedness of the primary tumour, since patients with right-sided primary tumours do not benefit from the addition of anti-EGFR to chemotherapy [8]. However, more recent data caution against the absolute use of this criterion [9, 10]. This stresses the need for further stratification and patient selection beyond *RAS/BRAF* mutations and tumour sidedness.

Recent work on molecular subtyping has demonstrated its predictive value for anti-EGFR therapies, and thus diagnostic utility in optimising selection criteria. The consensus molecular subtypes (CMSs) capture the biological heterogeneity in colorectal cancer by recognising four distinct subtypes [11]: with CMS1 characterised by microsatellite instability, strong immune activation and *BRAF*-mutations; CMS2 is an epithelial subtype with high chromosomal instability and prominent WNT and MYC signalling activation; CMS3 is also an epithelial subtype with metabolic dysregulation and is enriched for *KRAS*-mutations; and CMS4 represents a mesenchymal subtype with marked TGF-β activation and abundant stromal content. Whilst patients with epithelial CMS2/3 tumours benefit from anti-EGFR agents given as monotherapy or combined with an oxaliplatin-based chemotherapy regimen [12–14], patients with CMS4 tumours appear to have no or even a detrimental effect when anti-EGFR is added to an oxaliplatin-based regimen [13, 14]. In contrast, patients with mesenchymal CMS4 mCRC have a significant survival benefit when anti-EGFR is added to FOLFIRI, whereas for patients with CMS2 tumours no such benefit was seen [15]. An analysis on the differences between the CALB/SWOG 80405 and FIRE-3 studies also suggested that cetuximab activity is synergistic with irinotecan in all CMS subgroups, and with oxaliplatin only in CMS2 and CMS3 [16]. Based on these studies we hypothesised that anti-EGFR therapy has a subtype specific effect, which is dependent on the chemotherapy backbone used.

To further investigate the possible differential effect of backbone regimens in combination with anti-EGFR therapy for the different molecular subtypes we stratified patients enrolled in two prospective clinical trials, the COIN and the PICCOLO trials [17, 18]. The treatment effects of either a first-line oxaliplatin-based (COIN trial) or a second-line irinotecan backbone (PICCOLO trial) on anti-EGFR activity stratified by CMSs were studied. We hypothesised that anti-EGFR treatment is most effective when added to an oxaliplatin backbone in patients with CMS2/3 tumours in the COIN trial, while patients with mesenchymal CMS4 benefit most from anti-EGFR added to an irinotecan backbone as given in the PICCOLO trial. Furthermore, we performed exploratory analyses into differing effects according to tumour sidedness and CMSs to further define anti-EGFR therapy efficacy.

## Methods

### Patients

Details on both trial protocols have been previously reported [17, 18]. In short, the randomised controlled, multicenter, COIN trial was conducted between 2005 and 2008. Patients without previous chemotherapy for mCRC were randomly assigned to oxaliplatin and fluoropyrimidine chemotherapy (CAPOX (capecitabine and oxaliplatin) or FOLFOX (5-fluorouracil and oxaliplatin)), the same combination plus cetuximab, or intermittent chemotherapy. No selection based on mutation status was done.

The PICCOLO trial was a multicenter, randomised controlled trial in the second-line treatment of mCRC. Patients were included between 2006 and 2010 to a three-arm design of second-line therapy of irinotecan, irinotecan plus ciclosporin and irinotecan plus panitumumab. From June 10, 2008 panitumumab randomisation was restricted to patients with *KRASc.12,13,61* wildtype tumours.

Primary endpoint of both studies was overall survival (OS), with secondary endpoints of progression-free survival (PFS), tumour response (RECIST) and toxicity. For current analyses, only *RAS* (for both trials: *KRASc.12,13,61* and *NRASc.12,61*, additional for the PICCOLO trial: *KRASc.146* and *NRASc.13*) and *BRAFc.600* wildtype patients of the CAPOX/FOLFOX and CAPOX/FOLFOX with cetuximab treatment arms from the COIN trial and irinotecan and irinotecan with panitumumab arm from the PICCOLO trial were included.

Right-sided primary tumours were defined as tumours located proximal from the splenic flexure, left-sided tumours as tumours arising in or distal from the splenic flexure.

### CMS Classification

Tumour tissue from the primary tumour was collected for both trials from all available patients. For each primary tumour three or four cores were available on a tissue microarray (TMA) on a 4-µm-thick section slide. Tumours were stratified into the different consensus molecular subtypes using the previously developed immunohistochemistry (IHC)-based classifier [14, 19]. CMS1 patients were first classified using mismatch repair (MMR) protein expression status, identified by IHC of four markers (MLH1, MSH2, MSH6 and PMS2). Tumours with loss of expression of one of these markers were considered MMR deficient. Next, TMA slides were stained for five markers (CDX2, FRMD6, HTR2B, ZEB1 and KER) and classified into epithelial (CMS2/3) or mesenchymal subtype (CMS4) using the published image analysis pipeline and CMS-IHC classifier [14]. A probability of >60% was used for a core to be classified as mesenchymal, and a tumour was classified as CMS4 if at least one core was identified as mesenchymal.

### Statistical analysis

Stata version 15 was used for statistical analyses. Baseline patient characteristics were compared between the different subtypes using Pearson Chi-squared tests for categorical variables where the count was >5 in a cell and Fishers exact tests otherwise. Kruskal–Wallis tests were used for continuous variables. For calculation of *P*-values, unknowns were excluded.

Time-to-event curves for PFS and OS were calculated using the Kaplan–Meier method. Hazard ratios (HRs), 95% confidence intervals (CIs) and *P*-values were estimated using cox proportional Hazards models. Response data were compared between treatment groups using logistic regression and estimating odds ratios (ORs). All presented HRs and ORs for the main and sensitivity analysis are adjusted for age, sex and WHO performance status. *P*-values were two-sided and an arbitrary 5% cut-off was used for statistical significance. R software version 4.0.5 was used for *P*-value multiple testing correction using the Benjamini-Hochberg procedure, with an alpha of 0.05 [20]. We corrected *P*-values per independent analysis, i.e., for the total cohorts, left-sided and right-sided tumours. Adjusted *P*-values are indicated in the figure legends.

## Results

### Patient characteristics

**Fig. 1:**
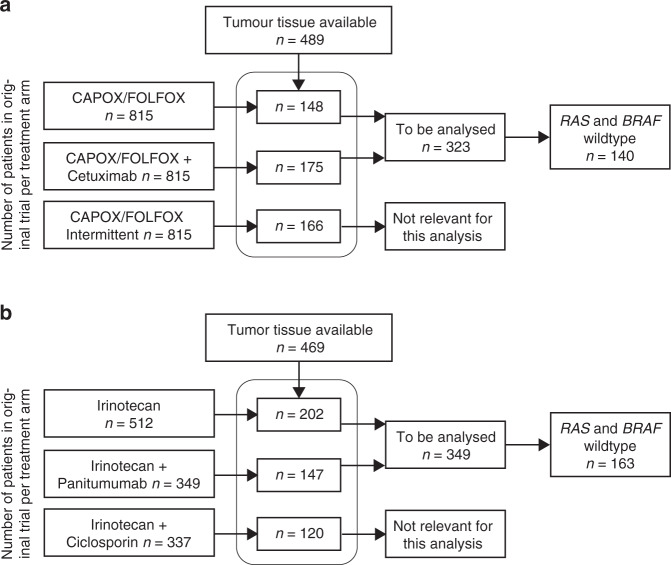
Study flow diagram. Overview of included samples for the COIN trial (**a**) and PICCOLO trial (**b**). CAPOX, capecitabine and oxaliplatin; FOLFOX, 5-fluorouracil and oxaliplatin.

Of 1630 COIN trial patients enrolled in the treatment arms of interest, tumour tissue for CMS classification was available for 323 patients (19.8%), of which 140 (43.3%) were *RAS* and *BRAF* wildtype. For the PICCOLO trial, of 861 patients enrolled in the treatment arms of interest for 349 (40.5%) tumour tissue was available for classification with 163 (46.7%) being *RAS* and *BRAF* wildtype (Fig. 1). All patients with tumour tissue available were classified into either CMS2/3 or CMS4 using a previously developed and validated immunohistochemical assay [14, 19]. For both cohorts the classified samples were representative for the total study population, but had improved PFS and OS (non-significant) with a higher proportion of resected primary tumours (Supplementary Table 1). This is inherently linked to the method of CMS classification used, as this requires sufficient tumour tissue for staining. In the *RAS* and *BRAF* wildtype cohort, the treatment arms were well balanced, apart from the primary tumour location in the PICCOLO trial, in which case the proportion of right-sided tumours was higher in the control (irinotecan) arm (Supplementary Table 2).

### CMS classification

In the classified wildtype cohort, 37% (52/140 COIN) and 31% (51/163 PICCOLO) of patients were classified as CMS4 (Table 1). CMS1 was excluded in the analysis due to low numbers in both cohorts (*n* = 4 and *n* = 2), in line with the notion that MMR deficient cancers are rare in mCRC [21]. Patients with CMS4 cancers were younger compared to CMS2/3 in the COIN trial (mean age 60.8 (SD = 10.2) versus 64.9 (SD = 8.4), *P* = 0.037), and CMS2/3 cancers were more frequently left-sided in the PICCOLO trial (45% versus 27%, *P* = 0.04) (Table 1). In the COIN trial CMS2/3 patients have a (non-significant) longer overall survival (22.3 versus 15.7 months, *P* = 0.07) compared to CMS4, for the PICCOLO trial on second-line anti-EGFR therapy there was no difference in survival between the epithelial and mesenchymal subtypes.

**Table 1:** Baseline and survival characteristics for the molecular subtypes in RAS and BRAF wildtype cohort.

Patient characteristic	Category	COIN				PICCOLO		
		CMS2/3 (*n* = 84)		CMS4 (*n* = 52)	*P*-value	CMS2/3 (*n* = 110)	CMS4 (*n* = 51)	*P*-value
Age^a^ (Mean (SD))			64.9 (8.4)	60.8 (10.2)	0.04	62.7 (10.3)	62.6 (10.3)	0.96
Sex No. (%)	Male		64 (76.2)	34 (65.4)	0.17	77 (70.0)	38 (74.5)	0.67
	Female		20 (23.8)	18 (34.6)		31 (28.2)	13 (25.5)	
	Unknown					2 (1.8)	0 (0.0)	
Performance status No. (%)	0		43 (51.2)	24 (46.2)	0.71	42 (38.2)	27 (53.9)	0.21
	1		36 (42.9)	23 (44.2)		61 (55.4)	22 (43.1)	
	2		5 (6.0)	5 (9.6)		7 (6.4)	2 (3.9)	
Primary tumour location No. (%)	Right		15 (17.9)	11 (21.2)	0.19	32 (29.1)	16 (31.4)	0.04
	Left		37 (44.0)	29 (55.8)		49 (44.6)	14 (27.4)	
	Rectum		32 (38.1)	12 (23.1)		25 (22.7)	21 (41.2)	
	Unknown		0 (0.0)	0 (0.0)		4 (3.6)	0 (0.0)	
Resected primary No. (%)	No		18 (21.4)	7 (13.5)	0.24	5 (4.6)	2 (3.9)	1.00
	Yes		66 (78.6)	45 (86.5)		104 (94.5)	49 (96.1)	
	Unknown		0 (0.0)	0 (0.0)		1 (0.9)	0 (0)	
Liver metastases No. (%)	No		25 (29.8)	8 (15.4)	0.06	28 (25.5)	16 (31.4)	0.50
	Yes		59 (70.2)	44 (84.6)		79 (71.8)	35 (68.6)	
	Unknown		0 (0.0)	0 (0.0)		3 (2.7)	0 (0.0)	
Lung metastases No. (%)	No		46 (54.8)	33 (63.5)	0.32	38 (34.6)	22 (43.1)	0.29
	Yes		38 (45.2)	19 (36.5)		70 (63.6)	28 (54.9)	
	Unknown		0 (0.0)	0 (0.0)		2 (1.8)	1 (2.0)	
Peritoneal metastases No. (%)	No		75 (89.3)	46 (88.5)	0.88	84 (76.4)	41 (80.4)	0.87
	Yes		9 (10.7)	6 (11.5)		22 (20.0)	10 (19.6)	
	Unknown		0 (0.0)	0 (0.0)		4 (3.6)	0 (0.0)	
Number of metastatic sites No. (%)	0/1		35 (41.7)	22 (42.3)	0.94	26 (23.6)	18 (35.3)	0.15
	2 or more		49 (58.3)	30 (57.7)		79 (71.8)	32 (62.7)	
	Unknown		0 (0.0)	0 (0.0)		5 (4.6)	1 (2.0)	
Outcome								
Overall survival (months)			22.3	15.7	0.07	13.2	12.7	0.26
Median (IQR)			(10.8–39.8)	(10.2–28.4)		(7.2–20.0)	(9.3–24.6)	
Progression-free survival (months)			9.1	9	0.17	5.3	5.6	0.26
Median (IQR)			(6.1–14.8)	(5.3–12.7)		(2.8–8.3)	(2.8–9.3)	
Best response No. (%)	CR or PR		58 (69.0)	36 (69.2)	0.80	29 (26.4)	13 (25.5)	0.83
	SD or PD		22 (26.2)	15 (28.8)		78 (70.9)	38 (74.5)	
	Unknown		4 (4.8)	1 (1.9)		3 (2.7)	0 (0.0)	

^a^Age at randomisation (years). Pearson Chi-squared test used for categorical variables where the count was >5 in a cell and Fishers exact test used otherwise Kruskal–Wallis test used for continuous variables. Unknowns were excluded for testing variables. Log-rank test was used for survival outcomes. CR or PR, complete or partial response; N, number of SD or PD, patients; SD, standard deviation; stable disease or progressive disease.

### Efficacy of anti-EGFR in CMS subtypes

**Fig. 2:**
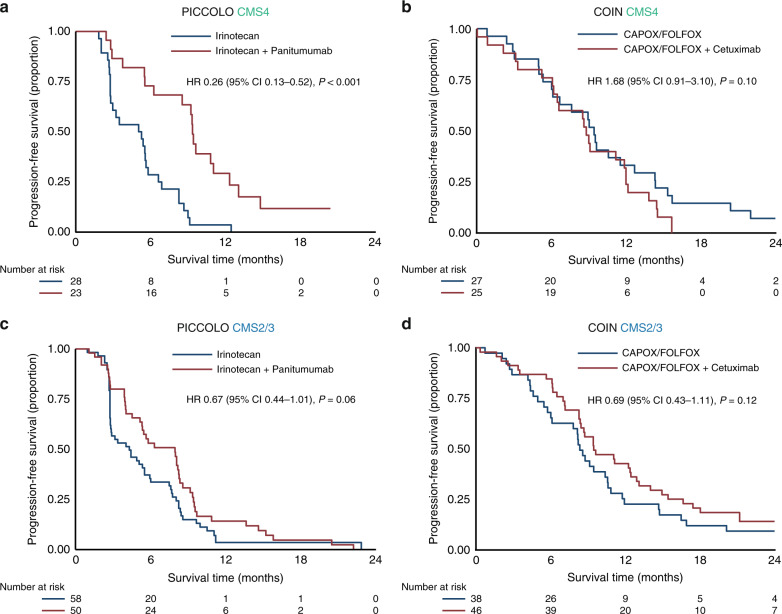
Molecular subtype-specific efficacy of anti-EGFR. Progression-free survival for CMS4 in the PICCOLO trial (**a**) and the COIN trial (**b**) and for CMS2/3 in the PICCOLO trial (**c**) and the COIN trial (**d**). HRs are adjusted for age, sex and WHO performance status. CAPOX capecitabine and oxaliplatin, FOLFOX 5-fluorouracil and oxaliplatin, HR hazard ratio. *P*-values adjusted for multiple testing: 0.12 (**a**), 0.10 (**b**), 0.12 (**c**), 0.004 (**d**).

When assessing the treatment response in the *RAS* and *BRAF* wildtype cohort there was a clear difference in response to anti-EGFR for the CMS4 tumours between both trials. When added to irinotecan there was a significantly longer PFS in CMS4 (HR 0.26, 95% CI 0.13–0.52, *P* < 0.001) (Fig. 2a), but when added to CAPOX/FOLFOX there was no beneficial effect of the addition of cetuximab (HR 1.68, 95% CI 0.91–3.10, *P* = 0.10) (Fig. 2b). For CMS2/3 patients there was also a (non-significant) benefit for anti-EGFR addition to irinotecan in the PICCOLO trial (HR 0.67, 95% CI 0.44–1.01, *P* = 0.06) (Fig. 2c), and a (non-significant) PFS benefit when added to CAPOX/FOLFOX in the COIN trial (HR 0.69, 95% CI 0.43–1.11, *P* = 0.12) (Fig. 2d). No difference in OS was observed.

As the combination of capecitabine-based chemotherapy and cetuximab was shown to be more toxic and, therefore, less effective compared to 5-fluorouracil-based chemotherapy and cetuximab [17, 22, 23], a sensitivity analysis for the COIN trial with patients receiving either CAPOX or FOLFOX was performed. Among wildtype patients, 63% (*n* = 86) was treated with either CAPOX or the combination of CAPOX with cetuximab. For CMS2/3 patients there was indeed a better outcome when cetuximab was added to FOLFOX, though this was not significant (PFS HR 0.45, 95% CI 0.17–1.20, *P* = 0.11; OS HR 0.48, 95% CI 0.17–1.31, *P* = 0.15) (Supplementary Fig. 1A).

Analysis of the response data convey comparable results, with response only observed in CMS4 when panitumumab was added to irinotecan (OR 8.52, 95% CI 1.69–43.05, *P* = 0.01) (Table 2). In CMS2/3 there was also a significantly better response when panitumumab was added to irinotecan (OR 4.27, 95% CI 1.66–11.00, *P* = 0.003), no difference was observed to the addition of cetuximab to CAPOX/FOLFOX (OR 1.72, 95% CI 0.60–4.95, *P* = 0.31).

**Table 2:** Best response^a^ for subtype-specific anti-EGFR efficacy.

Trial	Treatment	CMS2/3				CMS4			
		CR or PR No. (%)		OR (95% CI)	*P*-value	CR or PR No. (%)		OR (95% CI)	*P*-value
		Yes	No			Yes	No		
COIN	CAPOX/FOLFOX	23 (65.7)	12 (34.3)	1.00	0.31	19 (70.4)	8 (29.6)	1.00	0.95
	CAPOX/FOLFOX	35 (77.8)	10 (22.2)	1.72		17 (70.8)	7 (29.2)	1.05	
	+ Cetuximab			(0.60–4.95)				(0.28–3.96)	
PICCOLO	Irinotecan	9 (15.5)	49 (84.5)	1.00	0.003	3 (10.7)	25 (89.3)	1.00	0.01
	Irinotecan	20 (40.8)	29 (59.2)	4.27		10 (43.5)	13 (56.5)	8.52	
	+ Panitumumab			(1.66–11.00)				(1.69–43.05)	

^a^Best response: CR/PR versus SD/PD/Death. Only deaths which occurred within 12 weeks were included. ORs are adjusted for age, sex and WHO performance status. CAPOX, capecitabine and oxaliplatin (CAPOX/FOLFOX); CR, complete response; FOLFOX, 5-fluorouracil and oxaliplatin; OR, odds ratio; PR, partial response; SD, stable disease; PD, progressive disease.

### Effect of primary tumour location

In the combined *RAS* and *BRAF* wildtype cohort the majority of the tumours were left-sided (75%; COIN: 81% and PICCOLO: 69%). The distribution of the CMSs was similar in both trials and there was no difference between left- and right-sided tumours ((Left: CMS2/3 65%, CMS4 35%; Right: CMS2/3: 64%, CMS4: 36%; *X*^2^ = 0.077, *P* = 0.78) (Supplementary Table 3).

**Fig. 3:**
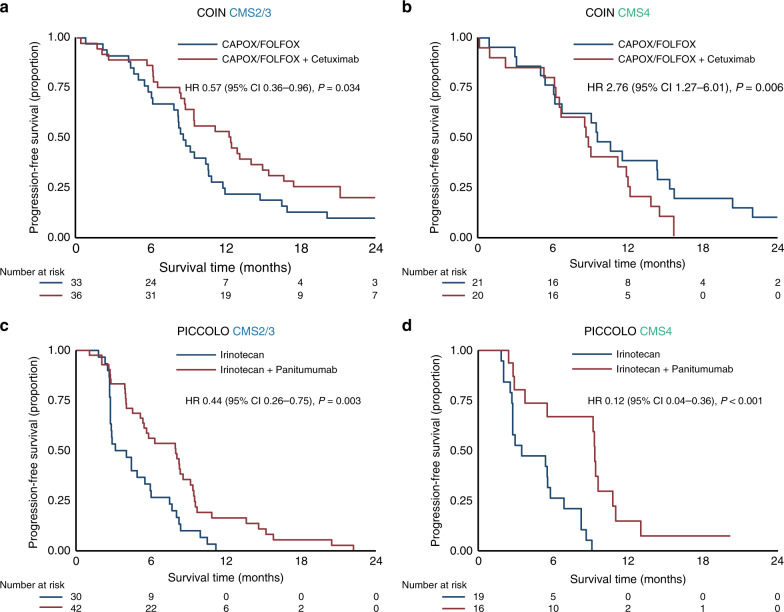
Molecular subtype-specific efficacy of anti-EGFR in left-sided tumours. Progression-free survival in the COIN trial for CMS2/3 (**a**) and CMS4 (**b**) and the PICCOLO trial for CMS2/3 (**c**) and CMS4 (**d**). HRs are adjusted for age, sex and WHO performance status. CAPOX capecitabine and oxaliplatin, FOLFOX 5-fluorouracil and oxaliplatin, HR hazard ratio. *P*-values adjusted for multiple testing: 0.034 (**a**), 0.008 (**b**), 0.006 (**c**), 0.004 (**d**).

In left-sided tumours, a clear difference between the subtypes in efficacy of anti-EGFR with the different backbones was observed, with CMS2/3 having a significant PFS benefit from the addition of anti-EGFR with both chemotherapy backbones (CAPOX/FOLFOX HR 0.57, 95% CI 0.36–0.96, *P* = 0.034; Irinotecan HR 0.44, 95% CI 0.26–0.75, *P* = 0.003). In CMS4 there was only a significant PFS benefit for anti-EGFR when added to irinotecan (HR 0.12, 95% CI 0.04–0.36, *P* < 0.001). When anti-EGFR was added to CAPOX/FOLFOX in CMS4 tumours even a detrimental effect was seen (HR 2.76, 95% CI 1.27–6.01, *P* = 0.006) (Fig. 3 and Supplementary Fig. 1B).

**Fig. 4:**
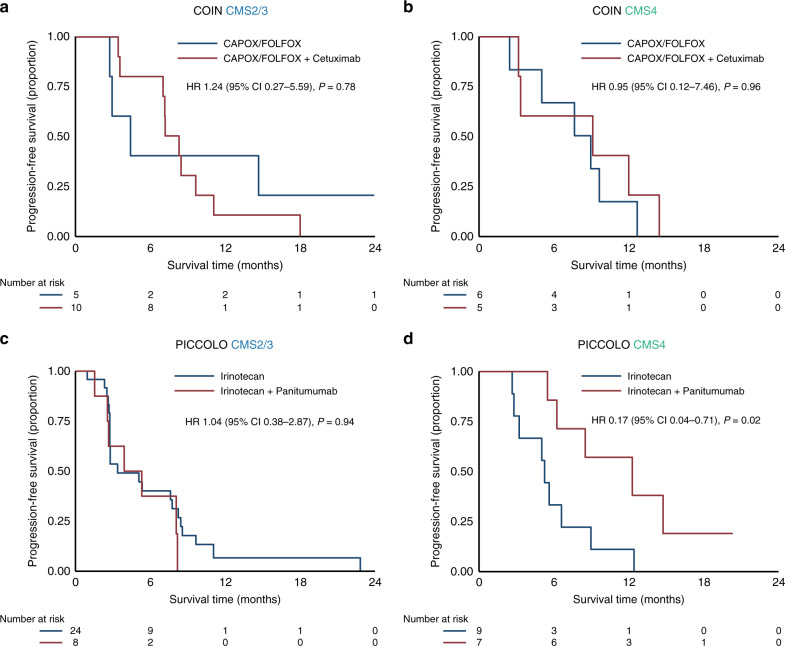
Molecular subtype-specific efficacy of anti-EGFR in right-sided tumours. Progression-free survival in the COIN trial for CMS2/3 (**a**) and CMS4 (**b**) and the PICCOLO trial for CMS2/3 (**c**) and CMS4 (**d**). HRs are adjusted for age, sex and WHO performance status. CAPOX capecitabine and oxaliplatin, FOLFOX 5-fluorouracil and oxaliplatin, HR hazard ratio. *P*-values adjusted for multiple testing: 0.96 (**a**), 0.96 (**b**), 0.96 (**c**), 0.08 (**d**).

For right-sided tumours a significant PFS and OS benefit was observed for CMS4 tumours when anti-EGFR was added to irinotecan as second line chemotherapy for both PFS (HR 0.17, 95% CI 0.04–0.71, *P* = 0.02) and OS (HR 0.20, 95% CI 0.05–0.83, *P* = 0.03) (Fig. 4 and Supplementary Fig. 1C). No benefit of anti-EGFR was observed in right-sided CMS2/3 tumours. A summary of our results with possible implications for treatment recommendations based on the CMSs and tumour sidedness is shown in Supplementary Fig. 2.

## Discussion

In this retrospective study of two trial cohorts, we show a differential effect of anti-EGFR therapy in the main CMS subtypes when combined with different chemotherapy backbones. The largest benefit of anti-EGFR was observed in patients with mesenchymal CMS4 tumours when combined with an irinotecan backbone. This effect was seen in both left and right-sided primary tumours. For epithelial CMS2/3 tumours there was a benefit from the addition of anti-EGFR when added to both oxaliplatin and irinotecan-based chemotherapy, which was only observed in left-sided primary tumours.

Our results are in line with others, who showed a PFS and OS survival benefit effect from anti-EGFR for CMS4 when added to an irinotecan-based first-line treatment regimen [15, 16]. The high disease control rate in CMS4 but also in CMS2 when anti-EGFR is combined with irinotecan was also shown in a recent publication, 93.3% and 76.9% for CMS2 and CMS4, respectively [24]. The CALGB/SWOG 80405 study did not show a survival difference for the addition of either bevacizumab or cetuximab to chemotherapy in CMS4 tumours [13]. Unfortunately, numbers to perform separate analyses on patients receiving either a FOLFOX or FOLFIRI chemotherapy backbone are relatively low.

Various clinical studies support the notion that the efficacy of anti-EGFR antibodies is restricted to left-sided tumours [8, 25, 26]. However, a recently updated meta-analysis indicated that anti-EGFR therapies could remain an option for patients with *RAS* wildtype right-sided tumours, as this significantly improved PFS and therapy response in both left- and right-sided tumours [9]. This finding is in line with the here reported results, which show that patients with both left- and right-sided CMS4 tumours benefit from the combination of irinotecan and panitumumab (Figs. 3 and 4). Therefore, anti-EGFR may potentially be considered in patients with right-sided CMS4 tumours.

Several preclinical studies have shown a synergistic effect between anti-EGFR therapy and irinotecan [27–29]. Tumour cell exposure to irinotecan leads to resistance through upregulation of EGFR signalling. Anti-EGFR overcomes this resistance through downregulation of the EGFR pathway which is upregulated by irinotecan [28, 30], explaining the benefit observed in irinotecan-refractory mCRC [31]. Another proposed mechanism underlying the enhanced tumour response of the combination of these two agents is a cetuximab induced suppression of mammalian heat shock protein 27 (HSP27), which is reported to be involved in irinotecan resistance, through blocking the JAK/STAT signalling pathway in RAS wildtype CRC cells [27].

There is some evidence that irinotecan-based regimens have a higher overall response rate (ORR) and improved PFS when combined with anti-EGFR in comparison to the combination with an oxaliplatin-based regimen [32, 33]. However, this may also be explained by the negative interaction between capecitabine in CAPOX regimens and anti-EGFR, as there is no difference in effectiveness when comparing the addition of anti-EGFR to either FOLFOX or FOLFIRI [22, 23].

Irinotecan-based treatment regimens, in general, appear to be more effective in CMS4 tumours when compared to oxaliplatin-based therapies [24, 34–36]. Moreover, preclinical studies in CMS4 cell lines and stem-like subtype tumours in patients, which are related to CMS4, show a marked response to topoisomerase inhibitors [37, 38]. However, the reason why mesenchymal tumours seem to be more susceptible to an irinotecan-based chemotherapy regimen is not yet understood. A possible mechanism of the antagonistic action of oxaliplatin in combination with anti-EGFR could be the fibroblast rich tumour microenvironment of CMS4 cancers, that increases the effects of oxaliplatin induced cytokines, which might subsequently antagonise the antitumor effects of cetuximab and the cetuximab-oxaliplatin synergy [16].

In the current study we show that patients with CMS2/3 tumours may benefit from anti-EGFR therapy combined with either an irinotecan or oxaliplatin backbone. The effects seen in the COIN trial were however small, which is likely explained by the fact that the majority of patients in this study were treated with CAPOX and cetuximab, notably this regimen is currently not recommended for its increased toxicity and lack of efficacy [17, 22, 23]. A sensitivity analysis showed a much better response when cetuximab was added to FOLFOX as compared to CAPOX (Supplementary Fig. 1A).

In both the COIN and PICCOLO trials a benefit of the addition of anti-EGFR was seen only for PFS, but not for OS, with an exception for the right-sided CMS4 tumours which benefited from the addition of anti-EGFR to irinotecan in both PFS and OS. The PFS is a direct measure of the treatment effect of the study, while OS is the result of the cumulative survival after all the lines of treatment that the patients underwent. Lack of overall survival benefit in the COIN trial was attributed by the authors to the combination of including patients with more advanced CRC and the more toxic effects of the combination of CAPOX and cetuximab [17]. For the FOLFOX treated patients we have indeed shown a trend towards OS benefit when anti-EGFR is added (Supplementary Fig. 1A). For the PICCOLO trial also only a PFS benefit was detected for the wildtype cohort. An inferior survival after relapse for the panitumumab treated patients was observed, especially for the population with a mutation (either *KRAS, BRAF, NRAS* or *PIK3CA*), possibly due to accelerated tumour growth caused by the anti-EGFR therapy [18]. In this study, however, we did see an OS benefit for the addition of anti-EGFR to panitumumab in right-sided CMS4 patients (Supplementary Fig. 1C).

We detected a trend towards OS benefit in CMS2/3 in the COIN trial, and no difference in survival between CMS2/3 and CMS4 in the PICCOLO trial. When looking at the total population, CMS4 confers the worst prognosis, because often these tumours present at a higher stage [21]. In mCRC the difference in survival between CMS2 and CMS4 is less pronounced, and worse survival is described for CMS1 [11]. Although numbers of CMS1 were low in this subpopulation, we did see indeed the lowest survival time for CMS1 in both trials (data not shown).

It is important to mention that the CMS classification of both studies was based on primary tumour tissue, which does not take into account any potential subtype switch during progression of disease, as well as the possible impact of chemotherapy on changing tumour biology as we classified the primary tumours pretreatment [39, 40].

Despite conducting this analysis on a smaller subcohort of the original trial due to limitations in tissue availability for classification, patient demographic characteristics and outcomes were consistent with the main trial analyses. The small survival benefit in both classified cohorts might be due to the higher number of resected primary tumours, which may be associated with improved OS [41]. This study analysed small subgroups and was underpowered to detect CMS/treatment interactions. Multiple comparisons were made and borderline significant results should be interpreted with caution. The retrospective nature of this study design and the cross-study comparison does not allow for definitive conclusions about the differential effect of the backbone therapy and anti-EGFR in the different subtypes, especially due to the different lines of therapy (first- versus second-line) which might contribute to the differences found. Nonetheless, we evaluated these two studies with a specific hypothesis in mind, which was confirmed by the result. This positions the current study as a starting point in refining selection criteria for anti-EGFR therapy based on CMS and primary tumour location. Additional studies are warranted to validate these findings and to reach definitive conclusions about the differential effect of chemotherapy backbones in relation with CMSs for anti-EGFR therapy. In addition, our study shows the potential clinical impact of stratifying patients into CMSs. This supports our current effort to implement the CMS classification into everyday clinical practice.

In conclusion, our results suggest that molecular subtypes may play an important role in the effectiveness of the type of chemotherapy backbone given in combination with anti-EGFR therapy. There is some evidence that patients with *RAS* and *BRAF* wildtype mesenchymal CMS4 tumours may only benefit when anti-EGFR is combined with irinotecan, even in right-sided primary tumours. For epithelial CMS2/3 tumours, we demonstrate no difference between irinotecan- and oxaliplatin backbones for the beneficial additive effect of anti-EGFR. This may provide the possibility of subtype-specific treatment strategies for the optimal use of anti-EGFR therapy.

## Supplementary information

Reproducibility checklist

Supplementary Material

## Data Availability

Data used in this study could be provided upon request, only with permission of the authors of the original studies.
