# Supplementary material for: Molecular subtype-specific efficacy of anti-EGFR therapy in colorectal cancer is dependent on the chemotherapy backbone
Source: Br J Cancer. 2021 Jul 12;125(8):1080–8. doi: 10.1038/s41416-021-01477-9 (PMC8505637; doi:10.1038/s41416-021-01477-9)

Supplementary Table 1. Baseline characteristics current cohort versus remaing patients[a]

	COIN		PICCOLO	
	**Current cohort**	**Remaining patients***	**Current cohort**	**Remaining patients***
**Total N(%)**	**323 (100.0)**	**1,307 (100.0)**	**349 (100.0)**	**512 (100.0)**
**Categorical variables**				
Received anti-EGFR N(%)	175 (54.2)	640 (49.0)	147 (42.1)	202 (39.5)
Female sex N(%)	112 (34.7)	449 (34.4)	117 (33.5)	159 (31.1)
Performance status 0 N(%)	160 (49.5)	590 (45.1)	140 (40.1)	231 (45.1)
Resected primary N(%)	253 (78.3)	612 (46.8)	327 (93.7)	293 (57.2)
Liver metastases N(%)	231 (71.5)	991 (75.8)	231 (66.2)	391 (76.4)
Lung metastases N(%)	139 (43.0)	524 (40.1)	194 (55.6)	278 (54.3)
Peritoneal metastases N(%)	46 (14.2)	195 (14.9)	95 (27.2)	108 (22)
>1 metastatic site N(%)	201 (62.2)	828 (63.4)	240 (68.8)	340 (69)
KRAS mutation N(%)	144 (44.6)	426 (43)	119 (34.1)	121 (28.7)
NRAS mutation N(%)	13 (4.0)	38 (3.9)	11 (3.2)	22 (5.8)
BRAF mutation N(%)	26 (8.1)	76 (7.8)	45 (12.9)	38 (9.1)
MSI N(%)	13 (4.5)	32 (4.4)	10 (9.1)	not available
**Site of disease**				
Right-sided N(%)	104 (32.2)	357 (27.3)	134 (38.4)	131 (25.6)
Left-sided N(%)	130 (40.2)	521 (39.9)	116 (33.2)	163 (31.8)
Rectum N(%)	88 (27.2)	409 (31.3)	92 (26.4)	211 (41.2)
Unknown N(%)	1 (0.3)	20 (1.5)	7 (2.0)	7 (1.4)
**Continuous variables**				
Age at randomisation (years) Mean (SD)	63.2 (9.3)	62.1 (9.9)	62.2 (10.6)	62.5 (10.5)
**Outcomes**				
Overall survival (months) Median (IQR)	18.6 (9.6-28.8)	15.4 (8.5-26.8)	10.5 (6.0-18.6)	9.2 (5.0-17.3)
Progression-free survival (months) Median (IQR)	8.3 (5.2-12.3)	7.9 (4.3-11.3)	4.5 (2.7-8.0)	3.0 (2.7-7.3)

[a]Total cohort of treatment arms of interest minus patients of the current cohort. Denominators for percentages are from the numbers of patients with data available on the covariate. IQR, interquartile range; N, Number of patients; SD, standard deviation.

Supplementary Table 2. Baseline characteristics in the *RAS* and *BRAF* wildtype cohort

		COIN			PICCOLO		
		CAPOX/ FOLFOX	CAPOX/ FOLFOX + Cetuximab		Irinotecan	Irinotecan + Panitumumab	
	Category	(N = 68)	(N = 72)	*P*-value	(N = 88)	(N = 75)	*P*-value
**Patient characteristic**							
Age[a] (Mean (SD))		63.2 (9.9)	63.0 (9.6)	0.91	61.9 (11.0)	63.1 (9.7)	0.56
Sex N(%)	Male	48 (70.6)	53 (73.6)	0.69	63 (71.6)	54 (72.0)	0.86
	Female	20 (29.4)	19 (26.4)		23 (26.1)	21 (28.0)	
	Unknown	0 (0.0)	0 (0.0)		2 (2.3)	0 (0.0)	
Performance status N(%)	0	30 (44.1)	38 (52.8)	0.30	40 (45.5)	30 (40.0)	0.79
	1	31 (45.6)	31 (43.1)		43 (48.9)	41 (54.7)	
	2	7 (10.3)	3 (4.2)		5 (5.7)	4 (5.3)	
Primary tumour location N(%)	Right	13 (19.1)	15 (20.8)	0.37	33 (37.5)	15 (20.0)	0.03
	Left	37 (54.4)	31 (43.1)		30 (34.1)	34 (45.3)	
	Rectum	18 (26.5)	26 (36.1)		21 (23.9)	26 (34.7)	
	Unknown	0 (0.0)	0 (0.0)		4 (4.5)	0 (0.0)	
Resected primary N(%)	No	12 (17.6)	14 (19.4)	0.79	4 (4.5)	4 (5.3)	1.00
	Yes	56 (82.4)	58 (80.6)		83 (94.3)	71 (94.7)	
	Unknown	0 (0.0)	0 (0.0)		1 (1.1)	0 (0.0)	
Liver metastases N(%)	No	18 (26.5)	19 (26.4)	0.99	24 (27.3)	20 (26.7)	0.98
	Yes	50 (73.5)	53 (73.6)		63 (71.6)	53 (70.7)	
	Unknown	0 (0.0)	0 (0.0)		1 (1.1)	2 (2.7)	
Lung metastases N(%)	No	40 (58.8)	42 (58.3)	0.95	32 (36.4)	28 (37.3)	0.94
	Yes	28 (41.2)	30 (41.7)		54 (61.4)	46 (61.3)	
	Unknown	0 (0.0)	0 (0.0)		2 (2.3)	1 (1.3)	
Peritoneal metastases N(%)	No	57 (83.8)	66 (91.7)	0.16	65 (73.9)	60 (80.0)	0.19
	Yes	11 (16.2)	6 (8.3)		22 (25.0)	12 (16.0)	
	Unknown	0 (0.0)	0 (0.0)		1 (1.1)	3 (4.0)	
Number of metastatic sites N(%)	0/1	26 (38.5)	32 (44.4)	0.46	19 (21.6)	25 (33.3)	0.09
	2 or more	42 (61.8)	40 (55.6)		66 (75.0)	47 (62.7)	
	Unknown	0 (0.0)	0 (0.0)		3 (3.4)	3 (4.0)	

[a]Age at randomisation (yrs). Pearson Chi-squared test used for categorical variables where the count was >5 in a cell and Fishers exact test used otherwise. Kruskal-Wallis test used for continuous variables. Unknowns were excluded for testing variables. N, Number of patients

Supplementary Table S3. Association between tumor sidedness and the molecular subtypes in the *RAS* and *BRAF* wildtype cohort. COIN trial (A). PICCOLO trial (B) and both trials combined (C).

A

	COIN		
	CMS2/3	CMS4	
Left-sided	69 (63%)	41 (37%)	110 (81%)
Right-sided	15 (58%)	11 (42%)	26 (19%)
	84 (62%)	52 (38%)	136 (100%)

Pearson Chi-squared test: $X^2 = 0.23$, df = 1, p-value = 0.64

B

	PICCOLO		
	CMS2/3	CMS4	
Left-sided	74 (68%)	35 (32%)	109 (69%)
Right-sided	32 (67%)	16 (33%)	48 (31%)
	106 (68%)	51 (32%)	157 (100%)

Pearson Chi-squared test: $X^2 = 0.023$, df = 1, p-value = 0.88

C

	Total		
	CMS2/3	CMS4	
Left-sided	143 (65%)	76 (35%)	219 (75%)
Right-sided	47 (64%)	27 (36%)	74 (25%)
	190 (65%)	103 (35%)	293 (100%)

Pearson Chi-squared test: $X^2 = 0.08$, df = 1, p-value = 0.78

Supplementary Figure 1. Molecular subtype specific efficacy of anti-EGFR in the *RAS* and *BRAF* wild-type cohort. CAPOX versus FOLFOX chemotherapy backbone in the COIN trial (A). Left-sided tumors in both trials and right-sided tumors in both trials (C). HRs are adjusted for age, sex and WHO performance status. CAPOX, capecitabine and oxaliplatin; CMS, consensus molecular subtype; FOLFOX, 5-fluorouracil and oxaliplatin; HR, hazard ratio. N, number of patients; PFS, progression-free survival; OS, overall survival.

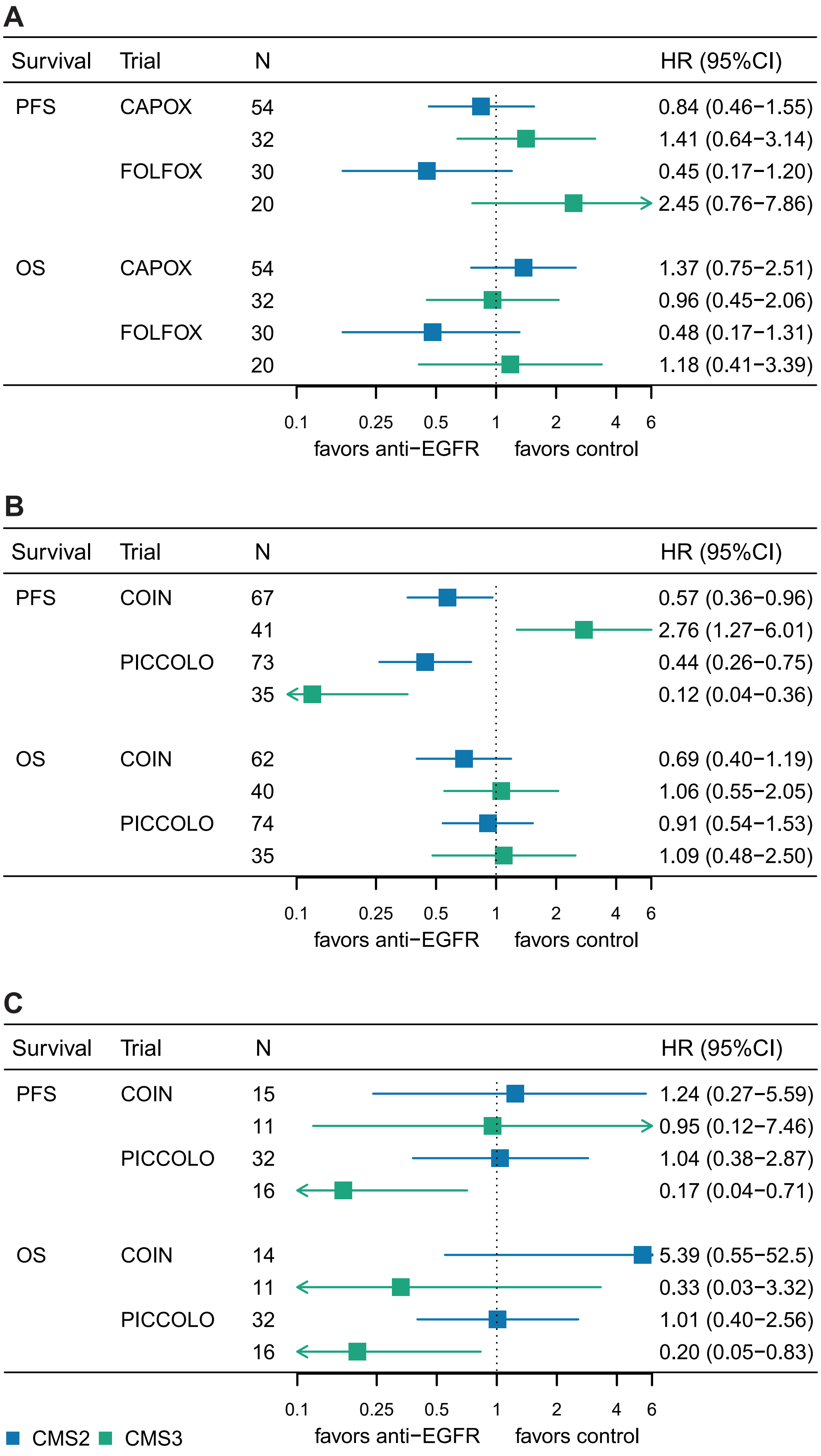

Supplementary Figure 2. Summary of our results with treatment recommendations based on the consensus molecular subtype and location of the primary tumor. CMS, consensus molecular subtype; FOLFOX, 5-fluorouracil and oxaliplatin; Left, left-sided primary tumor location; Right, right-sided primary tumor location.

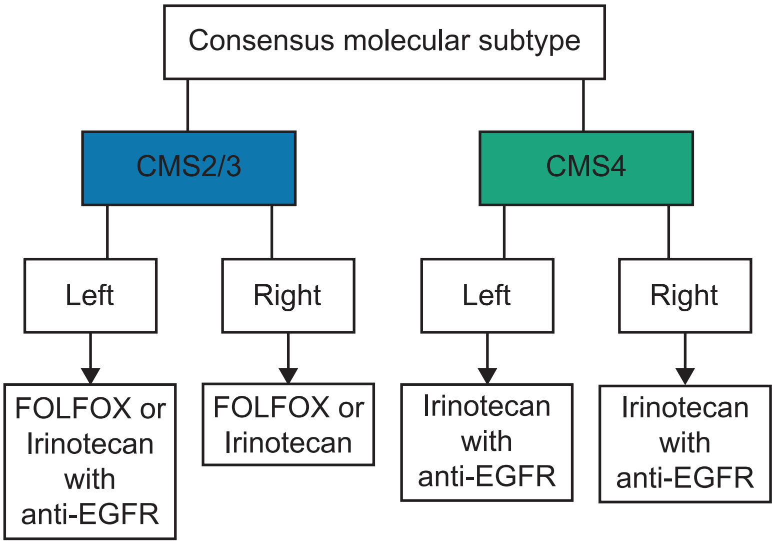

Supplement: Supplementary file 2 — Supplementary Material [file 41416_2021_1477_MOESM2_ESM.pdf]
